# Adherence to the 2015–2020 Dietary Guidelines for Americans Compared with the Mediterranean Diet in Relation to Risk of Prediabetes: Results from NHANES 2007–2016

**DOI:** 10.3390/nu15163546

**Published:** 2023-08-11

**Authors:** Pengcheng Wu, Lili Zhang, Yan Zhao, Miao Xu, Quan Tang, Guo-Chong Chen, Liqiang Qin

**Affiliations:** 1 Zhangjiagang Center for Disease Control and Prevention, 18 Zhizhong Road, Zhangjiagang 215600, China; 20215447005@stu.suda.edu.cn; 2 Department of Nutrition and Food Hygiene, School of Public Health, Suzhou Medical College of Soochow University, 199 Ren’ai Road, Suzhou 215127, China; 20214247012@stu.suda.edu.cn (L.Z.); kristinari@163.com (Y.Z.); gcchen@suda.edu.cn (G.-C.C.); 3 Yancheng Center for Disease Control and Prevention, 198 Kaifang Road, Yancheng 224001, China; xumiao9727@163.com (M.X.); tangquan_2008@126.com (Q.T.)

**Keywords:** prediabetes, Alternate Mediterranean Diet Index, Healthy Eating Index-2015, dietary pattern, dietary components

## Abstract

Prediabetes presents a high-risk state for the development of various diseases and is reversible by adhering to a healthy lifestyle. We conducted this analysis to explore the associations of the Healthy Eating Index-2015 (HEI-2015) and the Alternate Mediterranean Diet Index (aMed index) with the risk of prediabetes. The data were derived from the National Health and Nutrition Examination Survey, including 20,844 participants. Multivariable-adjusted odds ratios (OR) of prediabetes and 95% confidence intervals (CI) by tertile of diet quality scores were estimated using a weighted logistic regression. Compared to those in the lowest tertile, the multivariable-adjusted OR of prediabetes for the highest tertile was 0.82 (95% CI: 0.72, 0.94; *p* for trend = 0.005) for HEI-2015 and 0.87 (95% CI: 0.76, 0.98; *p* for trend = 0.02) for the aMed index. After mutual adjustment, the association for HEI-2015 (*p* for trend = 0.03) but not for the aMed index (*p* for trend = 0.59) remained significant. Among the component food groups and nutrients, higher intakes of red and processed meat, sodium, and total saturated fatty acids were associated with a higher risk of prediabetes, while moderate alcohol consumption was associated with a lower risk. In conclusion, adherence to the 2015–2020 Dietary Guidelines for Americans, as compared with the Mediterranean Diet, appeared to be more strongly associated with a lower risk of prediabetes among adults in the United States.

## 1. Introduction

Prediabetes, which is characterized by impaired glucose tolerance (IGT) and/or impaired fasting glucose (IFG) [1], represents a high-risk state for the development of diabetes, with an annual conversion rate of 5–10% [2], indicating that patients may progress to diabetes in the near future. Despite the absence of diabetes, it is also associated with a higher risk of cardiovascular disease, cancer, mental disorders, and mortality [3,4,5]. Today, prediabetes has become an increasingly concerning global public health issue that cannot be ignored.

Dietary elements are considered to be an important factor for diabetes prevention, especially so for at-risk individuals [6], but their applicability to the prevention of prediabetes remains unclear due to the limited research currently available. Some studies have shown associations between dietary intake, including red meat, alcohol consumption, and dietary protein, and the risk of prediabetes [7,8,9]. However, compared to examining individual food components or nutrients, the assessment of dietary patterns provides a holistic and comprehensive view of habitual food consumption and nutrient intake [10], which can also provide more reliable and practical dietary advice.

The Healthy Eating Index-2015 (HEI-2015) [11] and Alternate Mediterranean Diet Index (aMed index) [12] were chosen for analysis because of their global popularity and compatibility with the daily dietary habits of the American population. HEI-2015 evaluates the overall diet quality based on 13 components. The total score is a combination of the potentially beneficial components (including total fruit, whole fruit, total vegetables, greens and beans, whole grains, dairy, total protein foods, seafood and plant proteins, and fatty acids) and potentially detrimental components (including refined grains, sodium, added sugars, and saturated fats) and it emphasizes a variety of food groups and nutrient density [11]. The aMed index is a scoring system designed to assess adherence to the Mediterranean dietary pattern, and it evaluates the intake of major food groups, including fruit, vegetables, whole grains, nuts, legumes, fish, red and processed meat, and alcohol, in addition to the quality of dietary fatty acids reflected by the ratio of monounsaturated fatty acids to saturated fatty acids [12].

Both HEI-2015 and the aMed index have been extensively studied for their associations with various health outcomes [13,14,15]. However, their relevance to prediabetes risk has been much less explored, especially in a large, ethnically diverse, and nationally representative population. In this study, our aim was to determine whether adherence to two commonly recommended dietary patterns was similarly associated with the risk of prediabetes among adults in the U.S. National Health and Nutrition Examination Survey (NHANES). Although the two dietary patterns shared some similarities, such as emphasizing the intake of vegetables, fruits, and whole grains., there were differences in the components, scoring systems, and evaluation criteria. Therefore, we attempted to determine which dietary scoring system is more suitable for the prevention and management of prediabetes for the adult population in the U.S.

## 2. Materials and Methods

### 2.1. Study Population

NHANES is a program aimed at assessing the health and nutritional status of the U.S. population. The survey combines in-person interviews and physical examinations and uses a stratified, multistage probability approach to recruit a representative sample [16]. Our study used only publicly accessible data.

In our study, data from 2007 to 2016 surveys were considered due to the applicability to oral glucose tolerance test (OGTT) results during this period. Participants below 20 years of age were initially excluded. Subsequently, participants who self-reported pregnancy, had unreliable dietary recall data, did not meet the minimum criteria of NHANES dietary interview, or had a confirmed diagnosis of type 2 diabetes mellitus (T2DM) from a doctor or other health professional, insulin or oral hypoglycemic agent usage, fasting plasma glucose levels of 7.0 mmol/L or higher, 2 h postprandial plasma glucose levels of 11.1 mmol/L or higher, or glycosylated hemoglobin A1c (HbA1c) levels above 6.5% were also excluded. A total of 20,844 participants were included in the final analysis (Figure S1).

### 2.2. Dietary Assessment and Calculation of Diet Quality Scores

The current study focused on the first 24 h of dietary recall data obtained through in-person interviews [15,17]. Dietary assessment was conducted using data from NHANES and the Food Patterns Equivalents Database files from the United States Department of Agriculture (USDA) food composition database to calculate diet quality scores.

The components and scoring criteria of the HEI-2015 and aMed index are summarized in Tables S1 and S2, respectively, with higher scores indicating better diet quality. In brief, the 100-point HEI-2015 is a comprehensive system that comprises 13 distinct components, reflecting adherence to the 2015–2020 Dietary Guidelines for Americans. On the other hand, the 9-point aMed index, which is based on 9 components, assesses the consumption of food groups and nutrients to evaluate the degree of adherence to a Mediterranean-style diet.

### 2.3. Ascertainment of Prediabetes

Prediabetes was ascertained in accordance with the diagnostic criteria defined by the American Diabetes Association (ADA) [18]. Prediabetes was classified with fasting blood glucose (FBG) ranging from 5.6 to 7.0 mmol/L, a glucose level of OGTT ranging from 7.8 to 11.1 mmol/L, an HbA1c level ranging from 5.7 to 6.4%, or receiving a prediabetes diagnosis from a doctor or other health professional.

### 2.4. Assessment of Covariates

At baseline, sociodemographic characteristics, lifestyle factors, and body mass index (BMI) were collected through self-reporting from the standardized questionnaire data in NHANES. Ethnicity was classified into Non-Hispanic White, Non-Hispanic Black, Mexican American, and other ethnicities. Education was grouped as less than high school, high school or equivalent, and college or above. Marital status was grouped as married, previously married, and never married. Based on Liu’s report, the family poverty income ratio (PIR) was classified into three groups: ≤1.0, >1.0–3.0, and >3.0 [19]. Smoking status was grouped into three categories: “never smokers” for those who smoked less than 100 cigarettes in their lifetime, “current smokers” for those who were currently smoking, and “former smokers” for people who had smoked more than 100 cigarettes in their lifetime but were not currently smoking [20]. Drinking status was categorized into four groups: “never drinkers” for those who drank less than 12 cups in their lifetime, “former drinkers” for those who had drunk no more than 12 drinks in the past 12 months, “non-excessive drinkers” for those with an average daily alcohol consumption of less than 2 for men and less than 1 for women, and “excessive drinkers” for those who drank 5 or more alcoholic beverages at least one day on the same occasion (i.e., at the same time or within several hours) [21]. Physical activity (PA) was defined as the total metabolic equivalent (MET)-min/wk of vigorous work activity, moderate work activity, walking or bicycling, vigorous recreational activities, and moderate recreational activities according to the suggested MET scores. The levels were classified into three categories: low PA level (<600 MET-min/wk), moderate PA level (600–1500 MET-min/wk), and high PA level (>1500 MET-min/wk) [22].

### 2.5. Statistical Analysis

All statistical analyses were conducted using sample weights, strata, and primary sampling units to ensure accurate national estimates. The baseline characteristics of the study participants were reported based on their tertile of adherence to either the HEI-2015 or aMed index. Continuous variables with a normal distribution were compared among the three groups using one-way ANOVA tests, while continuous variables with a non-normal distribution were compared using the Kruskal–Wallis test. Categorical variables were analyzed using the chi-squared test. Multivariate logistic regression was employed to estimate odds ratios (ORs) and 95% confidence intervals (CIs) for prediabetes based on the tertile of the two diet quality scores and several dietary factors.

Three adjusted models were constructed to account for potential confounders. The first model was adjusted for sociodemographic variables (age, sex, ethnicity, education, marital status, and annual family income) and total energy intake (kcal/d). The second model further included smoking status, drinking status, and physical activity. The full model was additionally adjusted for BMI as it could be a potential mediator for the association between diet quality and prediabetes. To compare the two diet quality scores, we further adjusted them for each other in a separate exploratory model. Multiple imputation was conducted to minimize sample size reduction due to missing covariates.

Stratified analyses were conducted by age (<45 years, ≥45 years), sex (male, female), ethnicity (Non-Hispanic White, other), PIR (<1, ≥1), smoking status (current, never/former), drinking status (current, never/former), physical activity (≤600 MET-min/wk, >600 MET-min/wk), and BMI (<25 kg/m^2^, ≥25 kg/m^2^). The potential interactions were assessed by testing the corresponding multiplicative interaction terms.

We conducted a comprehensive analysis to further investigate the associations between specific food groups and nutrients that contribute to the evaluated diet quality indices and the risk of prediabetes. All information regarding food groups and nutrients was collected from the 24 h dietary recall. To ensure comparability, all individual dietary components (excluding alcohol) were standardized to account for a total energy intake of 1000 kcal/day [23]. The major dietary component was divided into tertiles, and alcohol consumption was grouped into three groups (<0 g, 0–25 g, and >25 g for males; <0 g, 0–15 g, and >15 g for females) [24].

We performed a set of sensitivity analyses to assess the robustness of our findings. These included the exclusion of participants with extreme energy intake (>6000 kcal/d or <800 kcal/d for men, >4000 kcal/d or <600 kcal/d for women) [25], those with self-reported hyperlipidemia and hypertension, as well as those with missing values across any of the covariates.

All statistical analyses were performed using R software (version 4.2.1). Statistical significance was considered for a two-sided *p*-value of less than 0.05.

## 3. Results

### 3.1. Participant Characteristics

Across five cycles spanning from 2007 to 2016, a total of 20,844 individuals were included in our present study. The baseline characteristics of the participants, categorized according to tertiles of the two dietary scores, are presented in Table 1. Individuals with a higher score of HEI-2015 or aMed index were more likely to be women, be of older age, have higher levels of education and family income, and be engaged in higher levels of physical activity, and exhibited healthier lifestyle behaviors such as nonsmoking, moderate alcohol consumption, and regular exercise. A substantial positive correlation existed between the HEI-2015 and aMed index, with a Pearson correlation coefficient of 0.64.

### 3.2. Diet Quality Scores and Risk of Prediabetes

**Figure 1:**
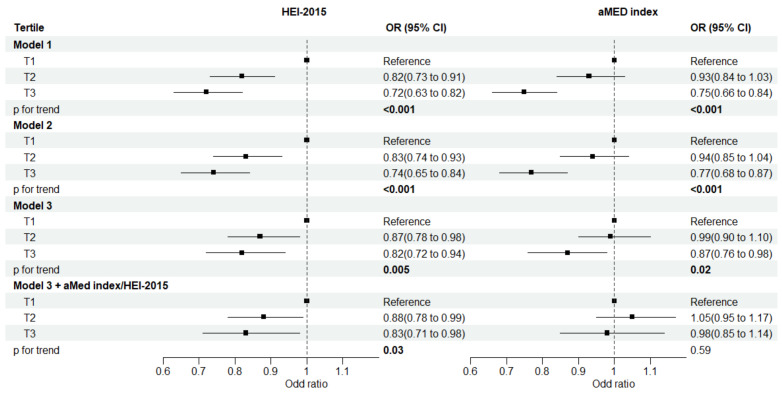
Relationship between diet quality scores and risk of prediabetes among US adults in NHANES 2007–2016. HEI, Healthy Eating Index; aMED, alternate Mediterranean diet. Model 1 was adjusted for age (years), sex, ethnicity (Non-Hispanic White, Non-Hispanic Black, Mexican American, other ethnicities), education (less than high school, high school or equivalent, college or above), marital status (married, previously married, never married), PIR (≤1.0, >1.0–3.0, >3.0), and total energy intake (kcal/d). Model 2 was adjusted for the covariates in model 1 plus smoking status (never, former, current), drinking status (never, former, non-excessive, excessive, only for HEI-2015), and physical activity (low, moderate, high). Model 3 was adjusted for the covariates in model 2 plus body mass index (kg/m^2^). Finally, both diet quality scores were mutually adjusted for each other in model 3. Odds ratio (OR) and 95% confidence interval (95% CI) were estimated using multivariable logistic regression models, taking into account the complex sampling design; *p* for trend values were calculated by assigning the median value to each tertile of diet quality score as a continuous variable.

Associations of the two diet quality scores with the risk of prediabetes are presented in Figure 1. After adjusting for sociodemographic variables and total energy intake, both diet quality scores were inversely associated with the risk of prediabetes. The results were slightly attenuated after further adjusting for lifestyle factors and BMI (Model 3). Comparing the highest with the lowest tertile, the multivariable-adjusted OR was 0.82 (95% CI: 0.72, 0.94; *p* for trend = 0.005) for HEI-2015 and 0.87 (95% CI: 0.76, 0.98; *p* for trend = 0.02) for the aMed index. In a separate exploratory model, we further adjusted for the mutual diet quality score to observe the stability of the two diet quality scores. The over-adjusted OR was 0.83 (95% CI: 0.71, 0.98; *p* for trend = 0.03) for HEI-2015 and 0.98 (95% CI: 0.85, 1.14; *p* for trend = 0.59) for the aMed index.

In stratified analyses, a protective effect against the risk of prediabetes was observed for the highest tertile of both dietary scores when compared to the lowest tertile across all subgroups (All *p* for interaction > 0.05, Table 2).

In sensitivity analysis, when participants with extremely high or low energy intake were excluded, the associations between dietary patterns and the risk of prediabetes remained largely unchanged (Table S3). Further adjustments for self-reported hyperlipidemia and self-reported hypertension did not substantially alter the results (Table S4). Consistent results were noted when participants with missing covariate data were excluded (Table S5).

### 3.3. Individual Food and Its Components and the Risk of Prediabetes

We further explored the association of major dietary components with the risk of prediabetes using data collected by the 24 h dietary recall in the NHANES (Table 3). After comprehensive adjustment for confounding factors, a comparison between the highest and lowest tertile revealed that a higher intake of red and processed meat was associated with an increased risk of prediabetes (*p* for trend = 0.016). In contrast, a lower risk of prediabetes was observed in men with moderate and high alcohol consumption (*p* for trend = 0.003) and in women with moderate alcohol consumption (*p* for trend = 0.050). For food components, higher intakes of total saturated fatty acids (TSFAT, *p* for trend = 0.005) and sodium (*p* for trend = 0.045) were both associated with a higher risk of prediabetes.

## 4. Discussion

In this cross-sectional study of nationally representative samples of U.S. adults, both HEI-2015 and the aMed index were significantly inversely associated with the risk of prediabetes. Notably, the risk of prediabetes associated with HEI-2015 was lower than that associated with the aMed index, and the associated risk remained significant for HEI-2015 but not for the aMed index in the separate exploratory analysis including both indices in the same model. This result highlights the stability of HEI-2015 in predicting the risk of prediabetes in U.S. adults. All stratified and additional analyses demonstrated the robustness of our study. Furthermore, red and processed meat, TSFAT, and sodium intake were positively correlated with the risk of prediabetes, while alcohol consumption showed the opposite relationship. 

Dietary patterns represent the diversity of diets, with HEI-2015 and the aMed index being widely recommended. The commonality of these two patterns focuses on a high intake of fruits, vegetables, whole grains, and dairy, as well as a lower intake of red and processed meat. Compared to unhealthy diets, adherence to recommended healthy dietary patterns is generally associated with reduced risks of many chronic diseases [24,26,27]. In particular, the relationship between dietary patterns and diabetes has been a focus of research. Previous studies have shown that high compliance with the Mediterranean diet can greatly reduce the risk of diabetes [28,29], and other evidence-based dietary patterns, such as the vegan and macrobiotic diet, Dietary Approaches to Stop Hypertension (DASH), and Alternative Healthy Eating Index (AHEI), can also reduce the risk of diabetes [30,31,32].

Individuals with diabetes often receive specialized dietary guidance from healthcare professionals and may adopt a rigorous approach to nutritional choices in their daily lives. Therefore, we focused on the population with prediabetes alone and investigated the association between dietary patterns and the risk of prediabetes. In contrast to our study, a small cross-sectional study involving 535 participants reported that neither the aMed index nor the aHEI dietary pattern was associated with the risk of prediabetes [33]. The discrepancy may be due to the smaller sample size and the limited range of the food frequency questionnaire (FFQ) used in that study. Regarding other dietary patterns, a cross-sectional analysis from the KORA FF4 study demonstrated that adhering to a Western diet was associated with a 92% higher risk of prediabetes compared to a prudent diet [34], suggesting that healthy dietary patterns can reduce the risk of prediabetes. In a longitudinal study in Rotterdam, a dietary pattern with a high intake of animal proteins from meat, fish, and dairy products was associated with a 35% increased risk of prediabetes [9]. As the main source of animal protein, we found that the intake of fish and dairy products did not affect the risk of prediabetes, while red meat and processed meat increased the risk of prediabetes. This is reasonable since some unhealthy components, such as saturated fatty acids, advanced glycation end products (AGEs), nitrates/nitrites, trimethylamine *N*-oxide (TMAO), branched amino acids (BCAAs), and endocrine disruptor chemicals (EDCs), in red and processed meat may contribute to the development of type 2 diabetes [35,36]. 

The associations between individual food or nutrient intake and prediabetes have been evaluated previously. Studies have discovered that red meat and TSFAT are positively correlated with the risk of prediabetes [37,38], which is consistent with our results. Our study observed, for the first time, a positive correlation between sodium intake and the risk of prediabetes. A few studies have provided indirect evidence of a positive correlation between sodium intake and the risk of diabetes [39,40]. Mechanistically, a diet high in TSFAT can induce glucose intolerance and insulin resistance [41], and excess sodium intake plays an important role in the development of inflammatory reactions [40], which are involved in the development of prediabetes and diabetes.

Our study found that alcohol consumption was significantly associated with a lower risk of prediabetes, especially in men. In a Swedish cross-sectional study, high alcohol consumption was correlated with an elevated risk of prediabetes, while high wine consumption was associated with a decreased risk among women [42]. Given the varying types and habits of alcohol consumption, as well as different classifications of drinking across different countries and regions, the defined ranges for alcohol intake can vary greatly, which can influence the results of alcohol consumption studies.

Our study has some strengths. To the best of our knowledge, it is the first study to investigate and compare the two commonly recommended dietary patterns (HEI-2015 and aMED) for their associations with risk of prediabetes, in a nationally representative sample (NHANES). In addition, we identified the key dietary components as major contributors to the benefits of the healthy dietary patterns. Furthermore, when assessing prediabetes, we employed multiple methods in combination, rather than relying solely on HbA1c, FBG, or OGTT criteria, which ensured a more accurate evaluation of prediabetes, minimizing potential ascertainment bias. However, several limitations of this study should be acknowledged. First, due to the observational nature of the NHANES study design, causal relationships cannot be established. Second, despite efforts to adjust for confounding factors, some residual or unknown confounding may still be present. Third, the dietary data from the questionnaire in NHANES may be subject to recall bias and may not accurately reflect long-term dietary habits. Lastly, the OGTT test was only performed in a subset of participants, preventing the distinction between IFG and IGT and limiting the ability to focus on subtypes of prediabetes.

## 5. Conclusions

Our study demonstrated that adherence to either HEI-2015 or the aMed index was significantly associated with a reduced risk of prediabetes. Furthermore, our findings suggest that HEI-2015 may be a more robust and reliable indicator compared to the aMed index for assessing the risk of prediabetes. These findings underscore the critical role of promoting healthy eating patterns as a crucial component of public health strategies aimed at preventing prediabetes and mitigating the risk of type 2 diabetes. However, further validation of our results in other large populations is warranted in future research.

## Figures and Tables

**Table 1:** Baseline participant characteristics according to tertile of diet quality scores among US adults in NHANES 2007–2016.

	HEI-2015			aMed Index		
	T1	T2	T3	T1	T2	T3
Age, years (mean (SD))	42.26 (16.01)	45.62 (16.41)	48.86 (16.81)	42.57 (16.08)	45.92 (16.67)	48.71 (16.61)
Sex = male (%)	52.2	49.6	43.2	51.7	49.1	43.1
Ethnicity (%)						
Non-Hispanic White	67.1	67.0	70.6	68.3	66.3	71.4
Non-Hispanic Black	12.9	11.3	7.7	12.7	11.0	7.6
Mexican American	8.8	9.1	6.9	8.3	9.1	7.0
other ethnicities	11.2	12.6	14.7	10.8	13.6	14.1
Education (%)						
Less than high school	18.9	15.5	11.7	20.3	15.3	9.4
High school or equivalent	27.2	22.4	15.8	27.6	22.4	13.7
College or above	53.8	62.1	72.6	52.1	62.2	76.9
Marital status (%)						
Married	58.1	61.2	66.5	57.5	61.6	67.8
Previously married	17.1	19.0	15.7	17.9	18.5	14.6
Never married	24.8	19.8	17.8	24.6	19.9	17.6
PIR (%)						
≤1.0	18.4	14.4	10.0	19.0	14.1	8.7
1.1–3.0	36.0	32.9	28.9	36.3	33.5	26.5
>3.0	45.6	52.7	61.2	44.7	52.3	64.8
Smoking status (%)						
Former	20.0	23.4	25.5	19.4	23.4	26.6
Current	30.2	20.9	12.1	32.3	19.2	10.4
Never	49.9	55.6	62.4	48.3	57.4	63.0
Drinking status (%)						
Nondrinkers	11.6	12.6	12.8	11.0	13.7	11.7
Former drinkers	14.6	12.3	10.7	13.4	13.1	10.5
Non-excessive drinkers	25.8	28.9	35.9	23.7	30.1	38.3
Excessive drinkers	47.9	46.2	40.7	51.9	43.2	39.4
Physical activity (%)						
Low	37.3	36.5	29.9	37.4	35.4	29.8
Moderate	13.1	14.5	18.0	13.2	14.9	18.2
High	49.6	48.9	52.1	49.4	49.7	52.1
Total energy intake, kcal/d (mean (SD))	2254.32 (1066.44)	2201.82 (991.77)	2058.64 (864.22)	2125.27 (1018.77)	2187.63 (1001.79)	2203.02 (894.91)
BMI, kg/m^2^ (mean (SD))	29.22 (7.07)	28.41 (6.39)	27.32 (5.74)	29.20 (7.01)	28.42 (6.34)	27.08 (5.76)

Data are weighted mean (SD) or weighted percentage (%). HEI, Healthy Eating Index; aMED, alternate Mediterranean diet.

**Table 2:** Stratified analyses of the associations between diet quality scores and risk of prediabetes among US adults in NHANES 2007–2016.

		HEI-2015				aMed Index			
		T1	T2	T3	P_interaction_	T1	T2	T3	P_interaction_
Age, years					0.134				0.832
<45	2669/10,121	1	1.09 (0.92, 1.30)	0.81 (0.66, 0.98)		1	1.12 (0.99, 1.28)	0.86 (0.70, 1.05)	
≥45	5852/10,723	1	0.78 (0.66, 0.92)	0.91 (0.77, 1.06)		1	0.94 (0.81, 1.09)	0.94 (0.80, 1.12)	
Sex					0.204				0.467
Male	4430/10,158	1	0.97 (0.83, 1.14)	0.81 (0.68, 0.97)		1	0.91 (0.78, 1.07)	0.89 (0.75, 1.06)	
Female	4091/10,686	1	0.78 (0.65, 0.93)	0.82 (0.70, 0.96)		1	1.05 (0.92, 1.20)	0.82 (0.69, 0.98)	
Ethnicity					0.060				0.169
Non-Hispanic White	3560/9252	1	0.81 (0.78, 0.84)	0.78 (0.73, 0.83)		1	0.94 (0.82, 1.06)	0.82 (0.78, 0.86)	
Other	4961/11,592	1	1.01 (0.88, 1.16)	0.91 (0.77, 1.06)		1	1.06 (0.92, 1.22)	0.97 (0.83, 1.14)	
PIR					0.326				0.783
<1.0	1635/4156	1	0.87 (0.76, 1.00)	0.82 (0.71, 0.95)		1	1.12 (0.90, 1.39)	0.82 (0.62, 1.08)	
≥1.0	6886/16,688	1	0.88 (0.71, 1.09)	0.84 (0.65, 1.09)		1	1.00 (0.89, 1.12)	0.84 (0.72, 0.97)	
Smoking status					0.271				0.171
Current	1803/4527	1	1.04 (0.86, 1.27)	0.78 (0.60, 1.03)		1	1.05 (0.87, 1.27)	0.99 (0.71, 1.38)	
Never/former	6718/16,317	1	0.83 (0.72, 0.96)	0.82 (0.71, 0.95)		1	0.99 (0.88, 1.11)	0.89 (0.77, 1.01)	
Drinking status					0.787				
Current	5571/14,605	1	0.86 (0.75, 1.00)	0.79 (0.68, 0.92)					
Never/former	2950/6239	1	0.91 (0.77, 1.08)	0.93 (0.77, 1.21)					
Physical activity					0.884				0.972
≤600 MET-min/wk	3654/8044	1	0.86 (0.74, 1.00)	0.88 (0.73, 1.04)		1	0.99 (0.84, 1.16)	0.95 (0.79, 1.14)	
>600 MET-min/wk	4867/12,800	1	0.87 (0.76, 1.03)	0.80 (0.67, 0.97)		1	0.98 (0.86, 1.12)	0.83 (0.70, 0.99)	
BMI					0.076				0.167
<25 kg/m^2^	1980/6726	1	0.97 (0.79, 1.19)	0.92 (0.73, 1.16)		1	0.94 (0.75, 1.17)	0.95 (0.75, 1.21)	
≥25 kg/m^2^	6541/14,118	1	0.82 (0.72, 0.94)	0.74 (0.64, 0.86)		1	0.96 (0.86, 1.08)	0.78 (0.68, 0.91)	

HEI, Healthy Eating Index; aMED, alternate Mediterranean diet. Data were adjusted for age (years), sex (female, male), ethnicity (Non-Hispanic White, Non-Hispanic Black, Mexican American, other ethnicities), education (less than high school, high school or equivalent, college or above), marital status (married, previously married, never married), PIR (≤1.0, >1.0–3.0, >3.0), total energy intake (kcal/d), smoking status (never, former, current), drinking status (never, former, non-excessive, excessive, only for HEI-2015), physical activity (low, moderate, high), and body mass index. The strata variable was not included in the model when stratifying by itself. The odds ratio (OR) and 95% confidence interval (95% CI) were estimated using multivariable logistic regression models, taking into account the complex sampling design; *p* for interaction (P_interaction_) values were calculated by testing the corresponding multiplicative interaction terms of the relevant covariates.

**Table 3:** Relationship between food components and risk of prediabetes among US adults in NHANES.

Components	Tertile for the Components			P_trend_
	T1	T2	T3	
Fruit	1	0.98 (0.87, 1.10)	0.94 (0.84, 1.07)	0.33
Vegetables	1	1.07 (0.98, 1.18)	0.99 (0.89, 1.09)	0.59
Whole grain	1	0.89 (0.80, 0.98)	0.93 (0.81, 1.08)	0.48
Refined grain	1	1.10 (0.99, 1.22)	1.07 (0.95, 1.20)	0.29
Dairy	1	1.04 (0.93, 1.17)	1.04 (0.92, 1.17)	0.55
Nut and seeds	1	0.96 (0.86, 1.07)	0.92 (0.83, 1.02)	0.12
Legumes	1	0.99 (0.88, 1.11)	1.00 (0.86, 1.16)	0.99
Red and processed meat	1	1.04 (0.94, 1.16)	1.13 (1.02, 1.26)	0.016
Fish	1	1.07 (0.92, 1.25)	1.00 (0.86, 1.16)	0.91
Fruit juice	1	1.01 (0.91, 1.13)	0.98 (0.86, 1.13)	0.78
Alcohol—male	1	0.78 (0.65, 0.94)	0.74 (0.61, 0.90)	0.003
Alcohol—female	1	0.74 (0.58, 0.94)	0.81 (0.65, 1.01)	0.050
Total saturated fatty acids	1	1.07 (0.96, 1.19)	1.19 (1.06, 1.33)	0.005
Total monounsaturated fatty acids	1	1.01 (0.91, 1.13)	1.10 (0.99, 1.23)	0.09
Total polyunsaturated fatty acids	1	1.08 (0.97, 1.21)	0.97 (0.88, 1.08)	0.46
Sodium	1	1.08 (0.96, 1.21)	1.14 (1.00, 1.30)	0.045
Added sugar	1	0.91 (0.82, 1.01)	1.10 (0.97, 1.24)	0.08

Data was adjusted for age (years), sex (female, male), ethnicity (Non-Hispanic White, Non-Hispanic Black, Mexican American, other ethnicities), education (less than high school, high school or equivalent, college or above), marital status (married, previously married, never married), PIR (≤1.0, >1.0–3.0, >3.0), total energy intake (kcal/d), smoking status (never, former, current), drinking status (never, former, non-excessive, excessive, only for HEI-2015), physical activity (low, moderate, high), and body mass index. The major dietary components were divided into tertiles, and alcohol consumption was grouped into three groups, including <0 g, 0–15 g, >15 g for female, and <0 g, 0–25 g, >25 g for male. Odds ratio (OR) and 95% confidence interval (95% CI) were estimated using multivariable logistic regression models, taking into account the complex sampling design; *p* for trend (P_trend_) values were calculated by assigning the median value to each tertile of individual dietary component as a continuous variable.

## Data Availability

The data used in this study are publicly available online (https://wwwn.cdc.gov/nchs/nhanes/; accessed on 9 August 2023).

## Supplementary Materials

The following supporting information can be downloaded at: https://www.mdpi.com/article/10.3390/nu15163546/s1, Figure S1. Flowchart of the study participants; Table S1. HEI-2015 components and criteria for scoring; Table S2. aMED index components and criteria for scoring; Table S3. Associations between diet quality scores and risk of prediabetes among US adults after excluding participants with extreme energy intake (>6000 kcal/d or <800 kcal/d for men, >4000 kcal/d or <600 kcal/d for women) in NHANES 2007–2016; Table S4. Associations between diet quality scores and risk of prediabetes among US adults with further adjustment of self-reported hyperlipidemia and self-reported hypertension in NHANES 2007–2016; Table S5. Associations between diet quality scores and risk of prediabetes among US adults after excluding participants with missing covariates in NHANES 2007–2016.
