# Supplementary material for: Adherence to the 2015–2020 Dietary Guidelines for Americans Compared with the Mediterranean Diet in Relation to Risk of Prediabetes: Results from NHANES 2007–2016
Source: Nutrients. 2023 Aug 11;15(16):3546. doi: 10.3390/nu15163546 (PMC10457824; doi:10.3390/nu15163546)
Supplement: Supplementary file 1 [file nutrients-15-03546-s001.zip › Table S1.pdf]

Table S1. HEI-2015 components and criteria for scoring.

Component	Dietary Constituents	Standard for Minimum	Min Score	Standard for Max	Max Score
Total Fruits	Total Fruit	No Fruit	0	≥0.8 cup eq. per 1000 kcal	5
Whole Fruits	Citrus, Melons, Berries + Other Intact Fruits	No Whole Fruit	0	≥0.4 cup eq. per 1000 kcal	5
Total Vege	Total Vegetables + Legumes (Beans and Peas) in cup equivalents	No Vegetables	0	≥1.1 cup eq. per 1000 kcal	5
Greens and Beans	Dark Green Vegetables + Legumes (Beans and Peas) in cup equivalents	No Dark-Green Vege or Legumes	0	≥0.2 cup eq. per 1000 kcal	5
Total Protein Foods	Total Meat, Poultry, and Seafood (including organ meats and cured meats) + Eggs + Nuts and Seeds + Soy + Legumes (Beans and Peas) in oz equivalents	No Protein Foods	0	≥2.5 oz eq. per 1000 kcal	5
Seafood and Plant Proteins	Seafood (high in n-3) + Seafood (low in n-3) + Soy + Nuts and Seeds + Legumes (Beans and Peas) in oz equivalents	No Seafood or Plant Proteins	0	≥0.8 oz eq. per 1000 kcal	5
Whole Grains	Whole Grains	No Whole Grains	0	≥1.5 oz eq. per 1000 kcal	10
Dairy	Total Dairy	No Dairy	0	≥1.3 cup eq. per 1000 kcal	10
Fatty Acids	(Total Monounsaturated Fatty Acids + Total Polyunsaturated Fatty Acids)/Total Saturated Fatty Acids	≤1.2	0	≥2.5	10
Moderation					
Refined Grains	Refined Grains	≥4.3 oz eq. per 1,000 kcal	0	≤1.8 oz eq. per 1000 kcal	10
Sodium	Sodium	≥2.0 g per 1,000 kcal	0	≤1.1 g per 1000 kcal	10
Added Sugars	Added Sugars	≥26% of energy	0	≤6.5% of energy	10
Saturated Fats	Total Saturated Fatty Acids	≥16% of energy	0	≤8% of energy	10
Total			0		100

HEI, Healthy Eating Index; Note: If the intake of one component meets the recommended minimum value, the score for that component is 0. If the intake meets the recommended maximum value, the score for that component is either 5 or 10 points. If the intake falls between the minimum and maximum values, the score is calculated using a linear interpolation formula.
