# Supplementary material for: Adherence to the 2015–2020 Dietary Guidelines for Americans Compared with the Mediterranean Diet in Relation to Risk of Prediabetes: Results from NHANES 2007–2016
Source: Nutrients. 2023 Aug 11;15(16):3546. doi: 10.3390/nu15163546 (PMC10457824; doi:10.3390/nu15163546)
Supplement: Supplementary file 1 [file nutrients-15-03546-s001.zip › Table S2.pdf]

Table S2. aMED index components and criteria for scoring.

Food Groups	Criteria for minimum score of 0	Criteria for maximum score of 1
Whole Fruit	< Median	≥ Median
Vegetables	< Median	≥ Median
Whole grain products	< Median	≥ Median
Legumes	< Median	≥ Median
Nuts	< Median	≥ Median
Fish	< Median	≥ Median
Red and processed meat	≥ Median	< Median
Ratio of monosat to sat lipids	< Median	≥ Median
Alcohol	< 5 or >15 g/d for female;<br>< 5 or >25 g/d for male	5-15 g/day for female;<br>5-25 g/day for male
Total	0	9

aMED, alternate Mediterranean diet;
