# Supplementary material for: Adherence to the 2015–2020 Dietary Guidelines for Americans Compared with the Mediterranean Diet in Relation to Risk of Prediabetes: Results from NHANES 2007–2016
Source: Nutrients. 2023 Aug 11;15(16):3546. doi: 10.3390/nu15163546 (PMC10457824; doi:10.3390/nu15163546)
Supplement: Supplementary file 1 [file nutrients-15-03546-s001.zip › Table S3.pdf]

Table S3. Associations between diet quality scores and risk of prediabetes among US adults after excluding participants with extreme energy intake (>6000 kcal/d or <800 kcal/d for men, >4000 kcal/d or <600 kcal/d for women) in NHANES 2007–2016.

	T1	T2	T3	P_{trend}
**HEI-2015**				
Median score (range)	37.450(9.500-44.542)	50.681(44.543-57.217)	65.100(57.218-95.997)	
Model1	1	0.81(0.72,0.90)	0.72(0.63,0.83)	<0.001
Model2	1	0.82(0.73,0.92)	0.74(0.65,0.85)	<0.001
Model3	1	0.87(0.77,0.97)	0.83(0.72,0.95)	0.01
**aMed index**				
Median score (range)	2(0-2)	3(3-4)	5(5-9)	
Model1	1	0.94(0.85,1.04)	0.75(0.66,0.84)	<0.001
Model2	1	0.96(0.86,1.06)	0.77(0.68,0.87)	<0.001
Model3	1	1.01(0.91,1.12)	0.87(0.76,0.99)	0.02

Model 1 was adjusted for age (years), sex(female, male), race (Non-Hispanic White, Non-Hispanic Black, Mexican American, other race), education (less than high school, high school or equivalent, college or above), marital status (married, previously married ,never married), PIR (≤1.0, >1.0-3.0,>3.0), and total energy intake (kcal/d). Model 2 was adjusted for covariates in the model 1 plus smoking status (never, former, current), drinking status (never, former, non-excessive, excessive，only for HEI-2015), and physical activity (low, moderate, high). Model 3 was adjusted for covariates in the model 2 and was additionally adjusted for body mass index (kg/m2). HEI, Healthy Eating Index; aMED, alternate Mediterranean diet; Odds ratio (OR) and 95% confidence interval (95% CI) were estimated using multivariable logistic regression models taking into account the complex sampling design; P for trend values were calculated by assigning the median value to each tertile of diet quality score as a continuous variable.
