# Supplementary material for: Adherence to the 2015–2020 Dietary Guidelines for Americans Compared with the Mediterranean Diet in Relation to Risk of Prediabetes: Results from NHANES 2007–2016
Source: Nutrients. 2023 Aug 11;15(16):3546. doi: 10.3390/nu15163546 (PMC10457824; doi:10.3390/nu15163546)
Supplement: Supplementary file 1 [file nutrients-15-03546-s001.zip › Table S4.pdf]

Table S4. Associations between diet quality scores and risk of prediabetes among US adults with further adjustment of self-reported hyperlipidemia and self-reported hypertension in NHANES 2007–2016.

	T1	T2	T3	P_{trend}
**HEI-2015**				
Median score (range)	37.450(9.500-44.542)	50.681(44.543-57.217)	65.100(57.218-95.997)	
Model3	1	0.87(0.78,0.98)	0.82(0.72,0.94)	0.005
Model3+self-reported hyperlipidemia	1	0.87(0.78,0.98)	0.82(0.72,0.94)	0.005
Model3+self-reported hypertension	1	0.88(0.78,0.98)	0.83(0.72,0.94)	0.006
**aMed index**				
Median score (range)	2(0-2)	3(3-4)	5(5-9)	
Model3	1	0.99(0.90,1.10)	0.87(0.76,0.98)	0.02
Model3+self-reported hyperlipidemia	1	0.99(0.90,1.10)	0.87(0.76,0.99)	0.02
Model3+self-reported hypertension	1	0.99(0.90,1.10)	0.86(0.76,0.98)	0.02

Model 3 was adjusted for age (years), sex(female, male), race (Non-Hispanic White, Non-Hispanic Black, Mexican American, other race), education (less than high school, high school or equivalent, college or above), marital status (married, previously married ,never married), PIR (≤1.0, >1.0-3.0,>3.0), total energy intake (kcal/d), smoking status (never, former, current), drinking status (never, former, non-excessive, excessive，only for HEI-2015), physical activity (low, moderate, high), body mass index. HEI, Healthy Eating Index; aMED, alternate Mediterranean diet; Odds ratio (OR) and 95% confidence interval (95% CI) were estimated using multivariable logistic regression models taking into account the complex sampling design; P for trend values were calculated by assigning the median value to each tertile of diet quality score as a continuous variable.
