# Supplementary figures and images for: Adherence to the 2015–2020 Dietary Guidelines for Americans Compared with the Mediterranean Diet in Relation to Risk of Prediabetes: Results from NHANES 2007–2016
Source: Nutrients. 2023 Aug 11;15(16):3546. doi: 10.3390/nu15163546 (PMC10457824; doi:10.3390/nu15163546)

**Figure S1.** Flowchart of the study participants.

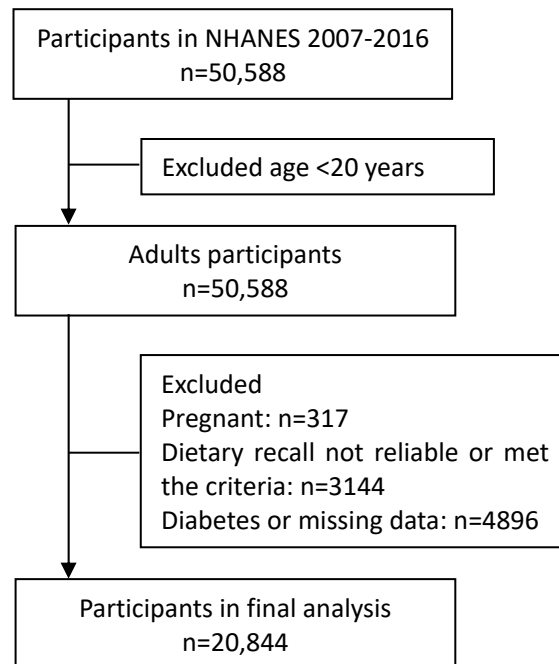

Supplement: Supplementary file 1 [file nutrients-15-03546-s001.zip › Figure S1.pdf]
